# Time from pre-eclampsia diagnosis to delivery affects future health prospects of children

**DOI:** 10.1093/emph/eox004

**Published:** 2017-02-08

**Authors:** Birgitte Hollegaard, Jacob A Lykke, Jacobus J Boomsma

**Affiliations:** 1 Centre for Social Evolution, Department of Biology, University of Copenhagen, Copenhagen, Denmark; 2 Department of Obstetrics, Rigshospitalet, Copenhagen, Denmark; 3 Faculty of Health Science, University of Copenhagen, Copenhagen, Denmark

**Keywords:** pre-eclampsia, public health, child morbidity, exposure *in utero*, metabolic syndrome

## Abstract

**Background and objectives:**

Pre-eclampsia often has detrimental health effects for pregnant women and their fetuses, but whether exposure in the womb has long-term health-consequences for children as they grow up remains poorly understood. We assessed overall morbidity of children following exposure to either mild or severe pre-eclampsia up to 30 years after birth and related disease risks to duration of exposure, i.e. the time from diagnosis to delivery.

**Methodology:**

We did a registry-based retrospective cohort study in Denmark covering the years 1979–2009, using the separate diagnoses of mild and severe pre-eclampsia and the duration of exposure as predictor variables for specific and overall risks of later disease. We analysed 3 537 525 diagnoses for 14 disease groups, accumulated by 758 524 singleton children, after subdividing deliveries in six gestational age categories, partialing out effects of eight potentially confounding factors.

**Results:**

Exposure to mild pre-eclampsia appeared to have consistent negative effects on health later in life, although only a few specific disease cases remained significant after corrections for multiple testing. Morbidity risks associated with mild pre-eclampsia were of similar magnitude as those associated with severe pre-eclampsia. Apart from this overall trend in number of diagnoses incurred across disease groups, hazard ratios for several disorders also increased with the duration of exposure, including disorders related to the metabolic syndrome.

**Conclusions and implications:**

Maternal pre-eclampsia has lasting effects on offspring health and differences between exposure to severe and mild pre-eclampsia appear to be less than previously assumed. Our results suggest that it would be prudent to include the long-term health prospects of children in the complex clinical management of mild pre-eclampsia.

## INTRODUCTION

Pre-eclampsia is part of a pregnancy specific syndrome defined clinically by new-onset hypertension and multi-organ failure; it complicates 5–8% of all pregnancies worldwide with potentially life threatening consequences for both mothers and children [1]. Pre-eclampsia mainly affects the liver, kidneys and central nervous system of expectant mothers and the symptoms may include, in addition to hypertension and proteinuria (protein-levels in urine exceeding 0.3 g/24 h), headaches and cerebral or visual disturbances which can progress into convulsions if the disorder proceeds to eclampsia.

There is general consensus on the optimal expectant management of severe pre-eclampsia before 34 weeks gestation [2, 3], where the risk of fetal prematurity is balanced against maternal risk of further progression of the disorder [2]. From 37 weeks onwards, prematurity of the baby is a minor concern and delivery is therefore usually induced when pre-eclampsia is severe and the same criterion is often also used for mothers with mild pre-eclampsia [4]. However, for gestations of intermediate duration (34–36 weeks) delivery decisions depend on individual obstetric management assessments, which may be informed by ongoing randomized clinical trials [5]. As immediate risks for damage to maternal health are fewer, pregnancies with mild pre-eclampsia are often subject to surveillance and carried to term when possible, both in Denmark and many other affluent countries.

The etiology of pre-eclampsia remains largely unknown and many have attempted to elucidate why a disease that is both damaging for mother and child does in fact exist. Pre-eclampsia has been shown to be long-term detrimental for maternal health as it increases risk of cardio-vascular diseases and type-2 diabetes later in life and incurs an increased likelihood of developing again in later pregnancies [6–8]. The standard medical interpretation of pre-eclampsia is that it represents an early maternal immunological maladaptation to ongoing fetal implantation, triggering a systemic response where the maternal endothelium produces the symptoms of hypertension and proteinuria [9]. This has been referred to as an example of the Goldilock principle, where the fetal implantation and inflammation signals cause symptoms when responses are too high or too low, but sustain normal pregnancies when in-between [10].

Evolutionarily hypotheses have been proposed as well to explain the conundrum of why pre-eclampsia has not been removed by natural selection despite the potentially severe impact on female reproductive success, especially in populations with natural birth rates before medical intervention and management was possible. First, pre-eclampsia has been suggested to be a by-product of selection for large brain size in the hominin lineage [11]. Endowing fetuses with a large brain is energetically expensive and has been associated with exceptionally deep trophoblasts invasion during implantation and early placenta development. If this early process partly fails the fetus will not receive sufficient maternal resources and may somehow induce elevated maternal blood pressure to compensate, leading to a higher likelihood of pre-eclampsia. According to this hypothesis, sufficient brain size at birth is assumed to be so important for future reproductive success that it precluded pre-eclampsia from disappearing from ancestral human populations [11, 12]. However, recent evidence for pre-eclampsia in chimpanzees appears to contradict this hypothesis [13, 14].

Alternatively, pre-eclampsia may be the result of an imbalance in the tug-of-war between paternal and maternal interests in resource provisioning to an unborn child. Trivers [15] recognized that maternal and offspring interests in resource provisioning are only partly aligned, so that offspring will tend to express traits to extract more resources than mothers are selected to provide when their life-time reproductive success is maximized by sharing resources equally amongst current and future offspring. Haig [16, 17] extended parent-offspring conflict theory by distinguishing between general age-dependent gene expression serving maternal or offspring interests, and genetic parental antagonism based on a special subset of imprinted genes affecting embryo and fetal provisioning in opposite directions. These imprinted genes are known from the placenta, where the paternal copy may be expressed and the maternal copy silenced or *vice versa*, as, for example, in IGF2 and its receptor [18]. Effects of such imprinted genes have been referred to as padumnal versus madumnal [19] or as patrigenic versus matrigenic [20].

Both parent-offspring theory in the sense of Trivers and parental antagonism in the sense of Haig are affected by the probability of parental promiscuity in the population, but in different ways. A higher probability of future maternal offspring being half siblings aggravates parent-offspring conflict because, from an offspring’s perspective, the relatedness difference between self (100%) and future full- (50%) or half-siblings (25%) is 2:1 and 4:1, respectively. However, when both parents are diploid, parental antagonism should be absent in populations with 100% monogamy. Paternal imprinting (silencing of maternal gene copies) is thus expected to be driven by the population-wide probability of serial monogamy or promiscuity, which imposes selection for compensating maternal imprinting (silencing of paternal gene copies) at other loci [16, 18, 21]. The parental antagonism hypothesis has gained considerable credibility in recent years because a number of imprinted genes with opposite effects on placental resource provisioning have been documented [22, 23]. In such cases, paternally imprinted genes have phenotypic effects to increase embryo or fetal provisioning, whereas maternally imprinted genes express phenotypes to resist such manipulation. As maternal blood pressure during pregnancy increases offspring provisioning, pre-eclampsia may thus be a byproduct consequence of this tug of war. However, we would expect maternal imprints to have evolved higher levels of control if pregnancy-related hypertension only had negative effects on maternal and offspring fitness.

In an earlier study, we provided a partial answer to the question of why pre-eclampsia may have been maintained in ancestral human populations by showing that mortality and morbidity in children of mothers with slightly elevated blood pressure in the first trimester were significantly lower relative to control pregnancies without any hypertension diagnoses. However, these correlations reversed when hypertension diagnoses continued during the second and third trimester [24]. This confirmed earlier smaller-scale studies that had pooled gestational hypertension diagnoses to show that modest hypertension may improve neonatal health provided it does not persist [25, 26]. These results suggest that padumnal demands are beneficial for offspring health provided madumnal compensation gains overall control so placentas develop normally. Pre-eclampsia could then be conceptualized as a negative byproduct when padumnal/madumnal balance is not achieved and trophoblast invasion remains underdeveloped, so that hypertension is the only mechanisms for paternally imprinted genes to secure additional provisioning in later trimesters [16, 17, 27]. When balanced, tug-of-war conflicts are expected to result in children of average size and weight at birth and above average health after birth, while imbalances to either side may produce babies that are small or large for gestational age with higher likelihoods of physical and mental health problems later in life [24, 28].

In the present study we used the same Danish public health data to address a complementary question that has so far remained unexplored, asking whether the duration of exposure to maternal pre-eclampsia may have lasting consequences for offspring health. This question is of interest both for the clinical management of pre-eclampsia, and evolutionarily because it explores the degree to which hypertension related disorders during pregnancy have a common cause as expected when unbalanced parental conflict drives the continued existence of these pregnancy disorders. Because recent research has indicated that the umbilical cord in pre-eclamptic pregnancies has increased atherosclerosis of the endothelium at birth [29], there are also proximate reasons for doing a large-scale study focusing on later child health, particularly to evaluate whether offspring cardiovascular diagnoses would correlate with the time from onset of maternal preeclampsia symptoms to delivery. We therefore designed a retrospective cohort study investigating all disease diagnoses except accidents from birth until 30 years of age in a large cohort of Danish children delivered after pregnancies with mild or severe pre-eclampsia versus normotensive controls, using gestational age and duration of diagnosed pre-eclampsia as predictor variables.

## METHODS

### Study population

We linked data from the Civil Registration System with the Danish National Patient Registry, Fertility Database, Birth Registry and Cause of Death Registry, via PNR numbers (anonymized personal identification numbers, CPR-numbers). We extracted information on all singleton births from 1 January 1979 to 31 December 2009, yielding 1 872 192 births by 1 004 129 mothers. We excluded births with missing values for one or more key variables such as PNR number, birth weight, birth length, gestational age, maternal birth-related diagnoses such as gestational diabetes, sequential births with short birth interval, child PNR numbers matching more than one mother, and pre-pregnancy diagnoses that could directly affect the risk of pre-eclampsia (any type of diabetes, purpura, circulatory diseases such as chronic hypertension, hypotension and ischemic heart disease, and insufficient kidney and liver function), which gave us a final cohort of 758 524 births by 413 594 mothers. Possible causes and consequences of caesarean sections could not be included in our analyses because they were not registered before 1996 and registration did not become diagnose-specific until 2004. The available data allowed us to extract information on all offspring diagnoses yielding 3 537 525 diagnoses across the maximally 30 years of follow-up. Offspring morbidity thus covered all diagnoses received through hospitalization and ambulant visits from birth to end of follow-up time (31 December 2009) with a minimum of 4 years of follow-up time.

In Denmark, medical services are free of charge and all residents have access to the same health care. This also applies to pregnant women, who are by default enrolled in a program with regular visits to a midwife and general practitioner, fetal ultrasound scans, advice on how to lose weight when deemed desirable, encouragement to give up smoking, etc. When registered as patients, all mothers and offspring in our study receive a medical evaluation and a diagnose code when referred or admitted to the hospital. These diagnoses are all registered (following ICD-8 from 1979 to 94 and thereafter ICD-10) in a hierarchical system with main disease categories, subcategories and further subcategories where appropriate. In our present study, morbidity refers to all diagnoses grouped primarily according to the 14 major disease groups defined by the WHO (see Supplementary Table S1) excluding only diagnoses related to accidents (injuries or other external causes: DS00-DZ99, DX60-DY09), which were not expected to have any relations to maternal pre-eclampsia. 

### Diagnoses, exclusions and gestation length categories

For statistical analyses, we categorized births into three groups based on diagnoses of pre-eclampsia (see Supplementary Table S1): mild pre-eclampsia (ICD 8/ICD 10 codes 63703/DO140), severe pre-eclampsia (ICD 8/ICD 10 codes 63704/DO141) and unexposed (controls). The Danish National Patient register has been validated by the Danish Health Authorities to ensure consistency of diagnoses for research across all years in the registries. These validations have investigated the diagnostic criteria behind all diagnoses from 1977 to 2010 and evaluated the use of both ICD-8 and ICD-10 codes to ensure that they are comparable. In this process the diagnoses of pre-eclampsia were matched between ICD-8 and ICD-10 codes so they corresponded to the set of diagnostic criteria in the guidelines from the Danish Health Authorities.

To ensure that data for fetuses exposed to both mild and severe pre-eclampsia would not be confounded across categories, we excluded women in the mild pre-eclampsia group who progressed into severe pre-eclampsia during the same pregnancy. We also excluded a residual group consisting of women diagnosed with unspecified pre-eclampsia (ICD 8 code 63709 and ICD 10 code DO149) due to inconsistencies in the diagnostic criteria, and women with any form of pre-eclampsia that progressed into eclampsia (ICD 8 code 63719 and ICD 10 code DO15) or the post-partum disease HELLP syndrome (ICD 8 code 63799 and ICD 10 code DO142). The final pre-eclampsia sample thus consisted of 28 471 births (23 920 with mild pre-eclampsia and 4551 with severe pre-eclampsia), while the unexposed cohort consisted of 704 013 births without any of these hypertensive disorders (Table 1).

**Table 1.** Base-line statistics, including means with SDs in brackets for all continuous variables, and absolute numbers with percentages in brackets for all categorical variables that were controlled for in the Model 1 (Fig. 2) and Model 2 + 3 (Fig. 3) analyses of the effects of exposure to mild and severe pre-eclampsia on child morbidity

Variables	Normotensive pregnancies	Pre-eclampsia pregnancies	
Degree of pre-eclampsia	Unexposed	Mild	Severe
Sample size (*N*)	704 013	23 920	4551
Maternal variables			
Age at delivery (SD)	28.6 (4.6)	27.7 (5.1)	28.0 (5.0)
Parity *N* (%)			
1	340 947 (48.4)	15 307 (64.0)	3192 (70.1)
2	267 079 (37.9)	5916 (24.7)	930 (20.4)
>3	95 977 (13.6)	2697 (11.3)	429 (9.4)
Education *N* (%)			
Primary education (10–12 years)	31 246 (4.4)	1360 (5.7)	241 (5.3)
Secondary education (12–15 years)	114 992 (16.3)	5365 (22.4)	906 (19.9)
Professional education	283 038 (40.2)	10 111 (42.3)	1929 (42.4)
B.Sc degree	215 401 (30.6)	5917 (24.7)	1210 (26.6)
M.Sc degree	56 003 (8.0)	1112 (4.7)	250 (5.5)
22+ years of education	3333 (0.5)	55 (0.2)	15 (0.3)
Paternal variables			
Age at delivery (SD)	31.3 (5.6)	30.4 (5.9)	30.7 (6.0)
Offspring variables			
Male *N* (%)	360 591 (51.2)	12 554 (52.5)	2363 (51.9)
Female *N* (%)	343 422 (48.8)	11 366 (47.5)	2188 (48.1)
Ponderal index adjusted for gestational age (SD)	14.40 (2.0)	14.36 (2.1)	13.60 (1.9)
Gestation age, days (SD)	279.3 (10.8)	276.7 (11.7)	258.5 (22.7)
Mean birth length, cm (SD)	52.1 (2.4)	51.8 (2.7)	48.5 (4.7)
Mean birth weight, gram (SD)	3544 (508.6)	3469 (607.1)	2753 (875.4)
Z-score (%) males/females^a^			
Z ≥ 2	2.3/2.4	5.3/5.2	3.3/2.7
2 > Z ≥ 1	12.4/12.9	14.5/13.4	8.9/6.9
−1 < Z < 1	71.2/71.1	60.0/59.3	47.3/42.6
−1 > Z ≥ −2	11.6/11.2	15.2/17.1	25.4/28.0
Z ≤ −2	2.5/2.3	5.1/5.0	15.2/19.8
Mean birth year	1995	1992	1994
Mean age at being diagnosed (years)^b^			
Infections	4.8 (0–6)	5.6 (0–8)	3.8 (0–5)
Neoplasm	11.4 (3–18)	13.0 (6–20)	10.0 (1–16)
Blood	6.6 (1–10)	7.1 (0–12)	5.4 (0–8)
Endocrine	9.2 (1–16)	11.1 (1–21)	6.8 (0–12)
Behavior	12.8 (6–18)	13.6 (8–19)	11.7 (5–17)
Nervous system	9.0 (2–14)	10.2 (2–17)	8.1 (1–13)
Eye/ear	5.1 (1–7)	5.7 (1–8)	4.6 (0–6)
Circulatory	13.1 (5–20)	15.6 (9–22)	12.3 (4–20)
Respiratory	4.1 (0–5)	4.9 (1–7)	4.0 (0–5)
Digestive system	8.6 (2–14)	9.6 (2–16)	6.9 (0–12)
Skin	8.8 (2–15)	10.6 (2–18)	9.2 (2–16)
Musculoskeletal	12.3 (6–18)	13.9 (9–19)	13.0 (8–18)
Genitourinary	11.1 (4–18)	12.4 (4–20)	10.5 (3–18)
Malformations	3.8 (0–6)	4.3 (0–7)	3.2 (0–5)
Gestation length categories *N* (%)			
Week 20–27 (391)	324 (0.1)	3 (0.01)	48 (1.1)
Week 28–33 (4663)	3539 (0.7)	181 (0.8)	683 (15.0)
Week 34–36 (20 249)	17 460 (2.9)	955 (4.0)	994 (21.8)
Week 37–38 (102 459)	92 859 (14.6)	4669 (19.5)	1203 (26.4)
Week 39–40 (410 475)	384 830 (58.3)	12 356 (51.7)	1206 (26.5)
Week 41–44 (217 056)	204 867 (30.8)	5753 (24.1)	352 (0.7)

a For birth weight, Z-scores (describing the distribution in standard deviations from the mean) were calculated based on the Danish reference growth curve [30].

b Mean age at diagnosis is given in years with first and third quartiles in brackets.

We stratified births into six categories based on gestational length: extremely preterm 20–27 weeks, very preterm 28–33 weeks, preterm 34–36 weeks, early term 37–38 weeks, full term 39–40 weeks and post-term 41–44 weeks. Due to low sample sizes in week 20–27 (unexposed: 391, mild pre-eclampsia: 3, severe pre-eclampsia: 48) we only included the results from this category in the supplementary material online and we refrained from detailed interpretations of the 28–33 weeks delivery cohort (unexposed: 4663, mild pre-eclampsia: 181, severe pre-eclampsia: 683) for the same reasons.

### Statistics and analyses

We used Cox proportional-hazard regression (R version ×64 2.15.3 (2013-03-01)—‘Security Blanket’, package Survival) to estimate hazard ratios and their confidence intervals, which correspond to the chance of being diagnosed at any point within a given time period relative to controls. When a hazard function *h*(*t*) is dependent on *n* covariates (*x*_1_, *x*_2_, …, *x_n_*), where the effect is measured by the size of the respective coefficients (*b*_1_, *b*_2_, …, *b_n_*), its mathematical expression becomes *h*(*t*) = *h*_0_(*t*) exp *b*_1_*x*_1_ + *b*_2_*x*_2_ + … + *b_y_x_y_*. Thus, *h*(*t*) is the probability (relative to controls) that an offspring at time *t* is diagnosed with a disease within one of the 14 disease groups. The changeover between ICD-8 and ICD-10 coding did not affect the diagnostic criteria, but the easier ICD-10 registration method increased the number of reported diagnoses. To compensate for this effect, we added the covariate ‘year’ to all our analyses to substantiate the general assumption that overall hazards remained constant through time.

We used three models based on Cox proportional-hazard regression. Model 1 is a combined analysis where both women with mild and severe pre-eclampsia were included, whereas models 2 and 3 are comparable multivariate analyses focusing on women with mild pre-eclampsia and women with severe pre-eclampsia, separately. The combined Model 1 analysis of hazard ratios associated with exposure to mild and severe pre-eclampsia partialed out the covariates maternal and paternal age at delivery, parity (first, second or third and above third birth), maternal education (primary, secondary, professional, B.Sc, M.Sc or above), sex of the child (male: 0/female: 1), number of days of pre-eclampsia (observed range 1–120; calculated from first day of diagnosis to delivery), year of delivery (to reduce noise from changes in total number of diagnoses—see above) and ponderal index (birth weight/(birth length^3^)) adjusted for gestational age (birth weight cut-offs 200 and 6500 g). For birth weight, Z-scores (birth weight—mean/SD) were calculated based on the national reference intrauterine growth curve for Denmark and are presented separately as these are often used in perinatological practices [30].

The two additional Cox regression analyses (Models 2 and 3) tested the extent to which child morbidity was affected by the duration of exposure to either mild or severe pre-eclampsia, because these separate effects have not previously been analysed. All covariates specified above for the Model 1 analysis were also included in the Model 2 and 3 analyses. We present the effects of exposure duration for mild and severe pre-eclampsia side-by-side to facilitate comparison, but the effects of the other covariates only for the combined Model 1 analysis as these effects remained very similar in the two additional models.

*P*-values were adjusted for multiple testing by FDR and we tested for homogeneity of variances for the hazard ratio response variables in each of the five classes of gestational age. There were no significant deviations from homogeneity, neither in the Model 1 analysis (mild PE: week 28–33: *P* = 0.95, week 34–36: *P* = 0.51, week 37–38: *P* = 0.13, week 39–40: *P* = 0.63, week 41–44: *P* = 0.37, severe PE: week 28–33: *P* = 0.64, week 34–36: *P* = 0.20, week 37–38: *P* = 0.12, week 39–40: *P* = 0.12, week 41–44: *P* = 0.28), nor the Model 2 and 3 analyses (mild PE: week 28–33: *P* = 0.09, week 34–36: *P* = 0.12, week 37–38: *P* = 0.13, week 39–40: *P* = 0.13, week 41–44: *P* = 0.47; severe PE: week 28–33: *P* = 0.06, week 34–36: *P* = 0.52, week 37–38: *P* = 0.07, week 39–40: *P* = 0.23, week 41–44: *P* = 0.50), which allowed comparison of the 14 disease groups across the 5 gestational age categories.

To elucidate overall morbidity risks of exposure to mild or severe pre-eclampsia, we performed Z-transformed combined probability tests with and without weighted sample sizes (R package survcomp 1.1.6) using the disease specific *P*-values. The weighted and unweighted samples sizes produced almost identical results, so we only report the former. Absolute risks of being diagnosed with diseases after exposure to either mild or severe pre-eclampsia, compared to the unexposed controls, were also calculated per gestation category and evaluated for differences with Mann–Whitney *U* tests using *P* < 0.05 as significance threshold. Model diagnostics and standard tests (based on Schoenfeld residuals) were performed in all analyses to check for violation of the Cox proportional-hazard regressions. No covariates showed any significant tendencies towards non-linearity.

We report means ± SD for a set of maternal and paternal covariates that potentially affect the incidence of mild or severe pre-eclampsia diagnoses and for a set of offspring response variables that are potentially affected by these maternal diagnoses. We then evaluate hazard ratios for child morbidity, again separately for exposure to either mild or severe pre-eclampsia and for the five (pre- to post-term) gestational age categories. We further evaluate the differential effects per disease and gestational age category for three offspring covariates (birth year, sex and ponderal index), four parental covariates (parity, father’s age, mother’s age and maternal education) and for the duration of exposure to pre-eclampsia, always comparing pre-eclampsia diagnoses with unexposed control pregnancies. Finally, we assess the isolated effect of duration of exposure to mild and severe pre-eclampsia (models 2 and 3) after partialing out the same set of covariates reporting the hazard ratio per day of exposure per disease class per gestational age category.

## RESULTS

### Child morbidity after exposure to maternal pre-eclampsia

**Figure 1.**
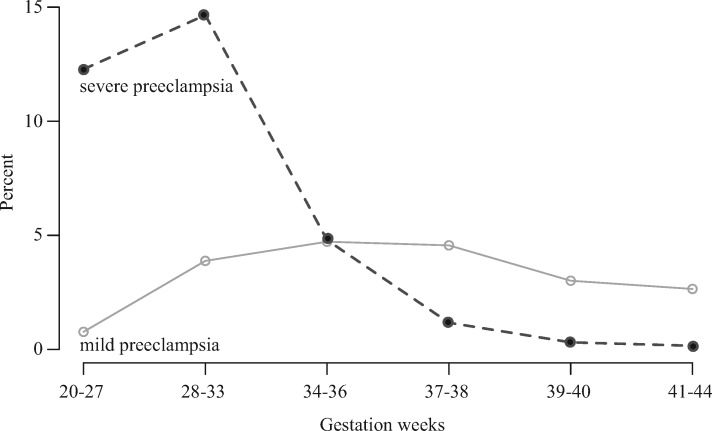
Percentages of Danish women with mild pre-eclampsia (open dots) or severe pre-eclampsia (black dots) diagnoses across the six time windows of delivery used in our analyses See Table 1 for sample sizes for both disorders

Week 34–36 was a turning point where mild pre-eclampsia became the more prevalent diagnosis at delivery after severe pre-eclampsia was the more common diagnosis in earlier deliveries (Fig. 1). Women diagnosed with any form of pre-eclampsia were younger (unexposed vs. mild PE: F_1, 727931 _= 932, *P* < 0.0001; unexposed vs. severe PE: F_1, 708562 _= 86.25, *P* < 0.0001), had lower parity (unexposed vs. mild PE: F_1, 727921 _= 1097, *P* < 0.0001, unexposed vs. severe PE: F_1, 708552 _= 484.2, *P* < 0.0001), and had fewer years of education (unexposed vs. mild PE: F_1, 727931 _= 1151, *P* < 0.0001, unexposed vs. severe PE: F_1, 708562 _= 101.6, *P* < 0.0001). Also the fathers were younger when a pregnancy was affected by pre-eclampsia (unexposed vs. mild PE: F_1, 727931 _= 592, *P* < 0.0001, unexposed vs. severe PE: F_1, 708562 _= 65.79, *P* < 0.0001) (Table 1).

Among the perinatal variables, the ponderal index of newborns adjusted for gestational age was slightly reduced after mild pre-eclampsia (unexposed vs. mild PE: F_1, 727907 _= 12.5, *P* = 0.0004) and substantially reduced after severe pre-eclampsia (unexposed vs. severe PE: F_1, 708538 _= 810.7, *P* < 0.0001) compared to children that were not exposed to pre-eclampsia (Table 1). The Z-scores for birth weight showed an excess of children 1–2 SD below average relative to unexposed children when mothers had been diagnosed with severe pre-eclampsia and more of these children were born prematurely, marginally so for mild pre-eclampsia (unexposed vs. mild PE: F_1, 727931 _= 1358, *P* < 0.0001) and by ca. 3 weeks for severe pre-eclampsia (unexposed vs. severe PE: F_1, 708562 _= 16 355, *P* < 0.0001) (Table 1). Further analysis of the seven covariates (see Supplementary Table S2) showed that: (i) A higher ponderal index is consistently associated with reduced morbidity in preterm and early term children. (ii) Boys tend to be more sensitive to neoplasm, endocrine and musculoskeletal disorders and girls to most other disease categories. (iii) As far as parity had a significant effect, later children tended to have slightly better health (reduced morbidities). (iv) Effects of paternal age were consistently non-significant, but older mothers (v) had slightly healthier children. (vi) A higher level of combined parental education was consistently associated with reduced child morbidity. (vii) As expected due to increases in number of diagnoses over the years, birth year significantly increased risk of being diagnosed.

**Figure 2.**
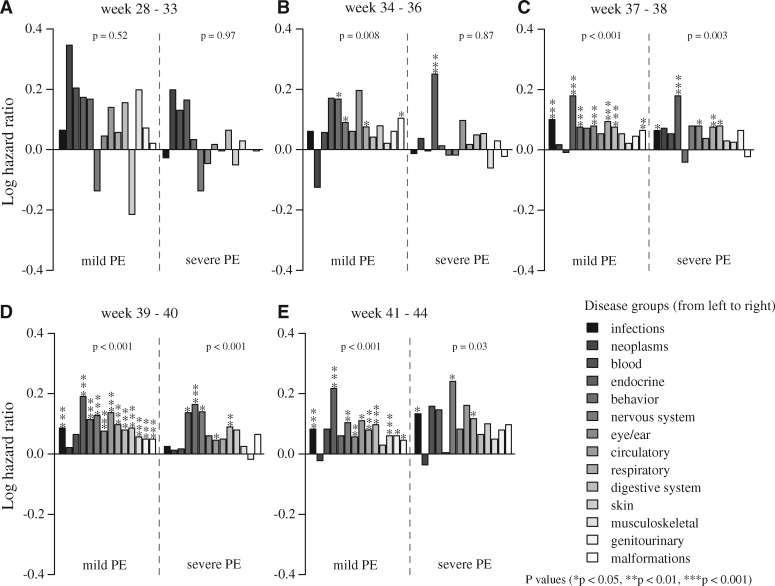
Risk (log hazard ratio) of being diagnosed up to 30 years after birth in each of the 14 disease groups (legend at the bottom right) for children born to mothers with either mild or severe pre-eclampsia (PE), relative to unexposed children born to normotensive mothers Logged hazard ratios represent increased (above 0) or decreased (below 0) risks. See Supplementary Table S5 for confidence limits. The gestation time period of 20–27 weeks was excluded due to low sample sizes (see text and Table 1 for details)

Offspring morbidity hazard ratios after pregnancies with either mild or severe pre-eclampsia were generally enhanced across all delivery time categories compared to children of non-hypertensive mothers (Fig. 2, see Supplementary Table S3), which was reflected in the combined *P*-values analysis, where only week 28–33 for mild and severe pre-eclampsia and week 34–36 for severe pre-eclampsia remained non-significant. Absolute risk estimates showed higher risks across all gestation categories when exposed to mild or severe pre-eclampsia compared to unexposed children. Children exposed to mild pre-eclampsia appeared to have consistently higher absolute risks than children exposed to severe pre-eclampsia, but this was only (marginally) significant for week 28–33 (*U *= 52, Z-score = 2.09, *P* = 0.04) and week 39–40 (*U *= 53, Z-score = 2.04, *P* = 0.04) (see Supplementary Table S4). The results for children born after mild or severe pre-eclampsia in the gestational age category of 28–33 and 34–36 weeks were less informative due to the smaller sample sizes (Table 1), but there appeared to be no difference in the magnitude of effects (bar heights in Fig. 2) across the last three delivery categories (>36 weeks) where sample sizes were larger. Neither did we find substantial differences between hazard ratios associated with mild and severe pre-eclampsia for the final three delivery categories, so the lower number of significant disorder-specific hazard ratios for severe pre-eclampsia can likely be attributed to the ca. five times lower sample sizes than for mild pre-eclampsia (Table 1).

The average ages of being diagnosed within the 14 main disease groups are concentrated in childhood and early adolescence, both for unexposed children and for children exposed to mild or severe pre-eclampsia (see Table 1 and Supplementary Fig. S1), indicating that our follow-up time was generally sufficient to capture the important effects.

### Increased child morbidity associated with duration of exposure to pre-eclampsia

**Figure 3.**
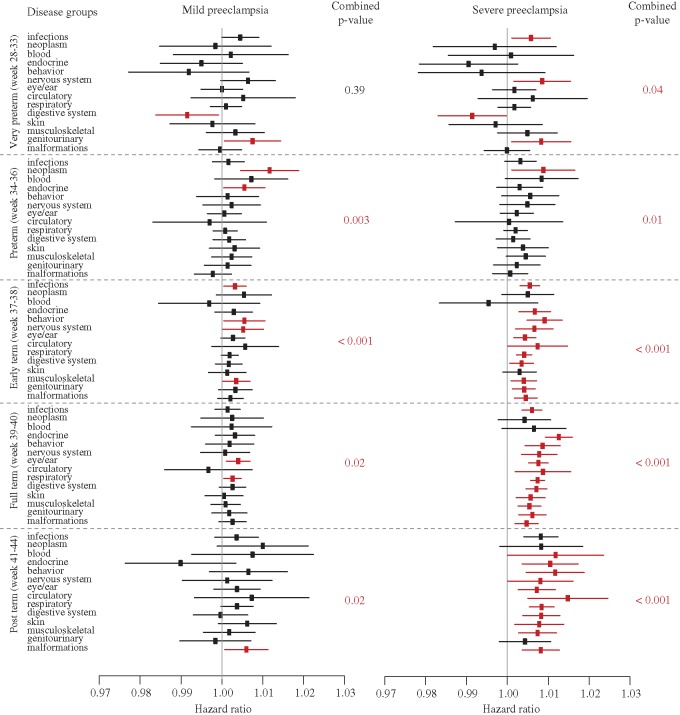
The effect of days of exposure to mild or severe pre-eclampsia (PE) after adjustment for categorical effects (diagnosis or not) and potentially confounding predictor variables (Table 1 and see Supplementary Table S2) on hazard ratios of child morbidity in five categories of gestational age, based on up to 30 years of follow-up Significant hazard ratios (HR) and their confidence intervals (CI) are plotted in red, but for mild pre-eclampsia all became non-significant after FDR adjustment, except for the neoplasm effect in week 34–36. For severe pre-eclampsia only digestive and genitourinary diseases in week 28–33, circulatory and genitourinary disorders in week 37–38 and blood and nervous system disorders in week 41–44 became non-significant after FDR adjustment. However, for mild pre-eclampsia exposure, the cumulative increases in hazard ratios across the 14 disease groups were all significant, except for the most premature deliveries (weeks 28–33). These cumulative *P*-values (calculated by a weighted Z-method of combined *P*-values and sample sizes) are given towards the right of each plot. A more detailed version of this figure with all case specific sample sizes, hazard ratios and the ±95% CIs is also provided (see Supplementary Table S5)

We found consistently increased risks per day of exposure to mild and severe pre-eclampsia (Fig. 3, see Supplementary Table S5), suggesting that exposure duration increases general morbidity during childhood and adolescence across most disease groups (also here the very preterm category of 28–33 weeks gestation is less informative for the mild pre-eclampsia pregnancies). Exposure to severe pre-eclampsia significantly increased relative risk of being diagnosed for almost all disease groups after week 37, whereas exposure to mild pre-eclampsia was associated with more modestly increased risks. Hazard ratios were above one in 12 out of 14 disease groups for deliveries at 34–36 weeks, in 13 of the 14 disease groups for deliveries in week 37–38 and week 39–40 and in 11 out of 14 disease groups for deliveries in week 41–44; respective overall significances *P* = 0.003, <0.001, 0.02 and 0.02 (Fig. 3). All but one (neoplasm in week 34–36) of the disease-specific *P*-values became non-significant after FDR adjustment, illustrating that an overall pattern across diseases can be strikingly consistent even though the composing disease-specific differences mostly fail to reach significance when considered in isolation. As aforementioned, the results presented in Fig. 3 (see Supplementary Table S5) were adjusted for all potentially confounding variables listed in Table 1.

### Analysing the specific effects of exposure to mild pre-eclampsia

Focusing on specific disease groups where a mild pre-eclampsia diagnosis both had a significant effect on general child morbidity (Fig. 2) and where the effect increased per day of exposure (Fig. 3, see Supplementary Table S5), the following results appeared to be most striking. First, children delivered preterm (gestation 34–36 weeks) had an increased risk of endocrine disorders (HR 1.48; 95% CI 1.17–1.87) compared to normotensive pregnancies (Fig. 2, see Supplementary Table S3), and the magnitude of this effect increased with days between diagnosis and delivery (HR 1.01; 95% CI 1.00–1.01) (Fig. 3, see Supplementary Table S5). Second, children delivered early term (gestation 37–38 weeks) had a significantly increased overall risk of mental and behavioral disorders (HR 1.19; 95% CI 1.04–1.37) and infections including parasites (HR 1.26; 95% CI 1.16–1.36) compared to normotensive controls (Fig. 2, see Supplementary Table S3) and also these risks increased significantly with days between diagnosis and delivery (HR 1.006; 1.001–1.011 and HR 1.003; 1.000–1.006, respectively) (Fig. 3, see Supplementary Table S5). Third, term-delivered children (gestation 39–40 weeks) had a significantly increased overall risk of eye/ear and adnexa disorders (HR 1.19; 95% CI 1.13–1.26) and respiratory system diseases (HR 1.25; 95% CI 1.21–1.30) compared to normotensive controls (Fig. 2, see Supplementary Table S3) and both these risks increased significantly with days between diagnosis and delivery (HR 1.004; 1.001–1.007 and HR 1.003; 1.000–1.005, respectively) (Fig. 3, see Supplementary Table S5). A similar double effect was found in post-term deliveries (gestation 41–44 weeks) for congenital malformations (HR 1.11; 95% CI 1.02–1.21 as main effect and duration of exposure as additional effect (HR 1.006; 1.001–1.011) (Fig. 3, see Supplementary Table S5).

## DISCUSSION

### The novelty and confirmative nature of our findings

To our knowledge, this is the first large-scale public health study on long-term morbidity in children associated with maternal diagnoses of pre-eclampsia and of the additional effects of duration of fetal exposure to pre-eclampsia. After conservative FDR adjustments, our results indicate significantly enhanced hazard ratios in the order of 10–25% for a series of disorders (Fig. 2, see Supplementary Table S3), with further increases in the order of 0.3–1.2% for every day of additional exposure to mild pre-eclampsia (Fig. 3). The effects outlined in the final paragraph of the results section could be ‘tips of icebergs’, because many similar but weaker trends were observed for other diseases, which were only significant when pooling effects across the 14 disease categories (Fig. 3, see Supplementary Table S5).

Our evaluation of the absolute risks point in the same direction as the risk patterns obtained from the hazard ratios showing consistently higher relative risk of morbidity across disease categories when exposed to either mild or severe pre-eclampsia compared to the unexposed group (Fig. 2, see Supplementary Table S4). The trends in hazard ratios are very similar when evaluating days of exposure (Fig. 3), a result that appears to justify pooling all pre-eclampsia diagnoses. However, this inference could only be obtained by doing the analyses separately as we have done here for the first time. The effects of severe pre-eclampsia are more likely to be statistically significant on a case-by-case basis in spite of the smaller sample sizes, but the effects of mild pre-eclampsia on morbidity later in life can apparently not be ignored, especially because duration of exposure (number of days diagnosed with the disease until delivery) is often longer than days exposed to severe pre-eclampsia. This underlines that possible long-term effects of exposure to mild pre-eclampsia deserve more specific study and clinical consideration because it is the more common disorder of the two.

Our results add relevant detail to a previous Danish registry study, which demonstrated increased morbidity in children up to three decades after pooled pre-eclampsia pregnancies [31]. That study specifically highlighted enhanced risks of endocrine, nutritional and metabolic diseases, which are all part of the metabolic syndrome that increases risks of cardiovascular diseases later in life. Our present results corroborate these earlier findings and suggests that part of these morbidity effects are associated with the duration of exposure.

The statistical effects of covariates that we uncovered largely confirmed previous insights for effects of ponderal index [30, 32] and education [33], and the tendency that older mothers have healthier children (see Supplementary Table S2) has also previously been documented [34]. As far as we evaluated the effects of severe and mild pre-eclampsia, we also confirmed known cumulative effects on size at birth [35].

### The relevance of the evolutionarily perspective

Pre-eclampsia has been described as ‘the disease of theories’ because its etiology has remained elusive. As we explained in the introduction, the enigmatic existence of the disorder may ultimately be due to an imbalanced resolution of evolutionarily conflict between patrigenic (padumnal) and matrigenic (madumnal) effects on fetal provisioning [12, 16, 17, 24], with pre-eclampsia as a collateral damage byproduct. The rationale would then be that the expression of conflict by itself increases the probability of offspring attaining above average health, but that this favorable outcome is conditional on a controlled balance between paternal and maternal provisioning being reached early in gestation, because less common unbalanced outcomes are clearly pathological [24].

The alternative ultimate hypothesis driven by the need to sustain early human brain development [11, 12] and the necessity for inflammation signals during implantation to be balanced according to the Goldilock Principle [10] is less general and inconsistent with pre-eclampsia also occurring in great apes [13, 14]. Chimpanzee pre-eclampsia does not, however, compromise the parental antagonism hypothesis as promiscuity in our closest primate relatives is higher than in humans [36]. If the continued existence of pre-eclampsia is indeed driven by factors that depend on the combination of maternal and paternal genes rather than on maternal genes only, an important implication of our present and previous study [24] might be that searching for a heritable component (maternal genes pre-exposing to pre-eclampsia) is unlikely to be successful.

### Proximate mechanisms mediating pre-eclampsia and the clinical relevance of our results

It has recently been suggested that pre-eclampsia may be mitigated by the peripheral T regulatory cells (pTreg) that evolved to provide a stronger maternal-fetal tolerance in placental mammals [37]. As pre-eclampsia always involves elevated maternal blood pressure, it is interesting that children of mildly pre-eclamptic mothers have substantially increased risks of endocrine disorders, including diabetes and developing elevated blood pressure problems themselves later in life [31, 38]. The extent to which these effects could be due to recent changes in human life-style remains to be investigated.

It is known that the mild pre-eclampsia syndrome may be recurrent across pregnancies of the same women with negative implications such as fetal growth restriction and placental abruption [7], comorbidities that often dictate expeditious delivery because they are known to be associated with increased child morbidity [39]. Prematurity *per se* is also linked to later learning difficulties in basic school (age 6–15) and risk of not completing an education [40], so exposure to pre-eclampsia combined with fetal growth restriction is a known aggravating factor with respect to later performance parameters [41].

The organ systems most sensitive to mild pre-eclampsia exposure appear to be the neurological system, eyes and ears and the respiratory system (Figs 2 and 3, see Supplementary Tables S3 and S5). The respiratory system effect has previously been linked to caesarean sections [42], which could confound our inferences for these risks as caesarean sections are more common for pre-eclampsia deliveries [43]. The neurological effects may be associated with the diagnosed mental and behavioral disorders and with retinopathy [44], for which we found exposure duration effects in early-term deliveries (Fig. 3). These aspects would benefit from further investigations into specific diagnoses to disentangle where the highest risks present themselves, because these details remain unresolved in the grouped diagnoses of our present study.

For clinical practice our results imply that the management of mild pre-eclampsia by expeditious delivery after 37 weeks may reduce life-time morbidity while also preventing progression of maternal perinatal disease—inferences that appear to match the results from the seminal HYPITAT trial [4] (but see below for some caveats). The gradual build-up of these later morbidity risks from gestation weeks 34–36 onwards thus suggests that future health prospects of newborns should become part of the clinical management of pre-eclampsia from at least 37–38 weeks gestation and possibly earlier (week 34–36) (Fig. 1).

### Caveats

Because of the large sample sizes, our cohort study based on a complete census of all live births and subsequent medical diagnoses in Denmark during three decades allowed for sufficient statistical power to detect small effects. As hospitalizations are most common before age 10 and after age 45 (except for pregnancy-related reasons and deliveries [45]) and our data confirmed that diagnoses are concentrated in childhood and early adolescence for all disease groups (Table 1, see Supplementary Fig. S1), we believe that the follow-up of 4–30 years that was available to us should generally have been appropriate for assessing the morbidity consequences of exposure to pre-eclampsia. However, we realize that this follow-up time remains less than ideal for metabolic syndromes as these tend to be diagnosed later in life than behavioral and mental disorders [28].

Although we could partial out more potentially confounding effects than most other public health studies, we had no data on maternal body mass index and smoking, factors that have previously been shown to increase the risk of pre-eclampsia [46], and we did not know which children had been born after C-section (see Methods). It is important to note that socioeconomic variables in Denmark are expected to have smaller effects on general health than in most other countries, because free health care is available to all residents and the country’s GINI coefficient of socioeconomic inequality is among the lowest in the world [47]. There is a tendency that Danish families of lower socioeconomic status have a higher degree of encounters with hospitals, while families of higher socioeconomic status have more encounters with their general practitioner [45] but this kind of effect tends to account for less than 1% of the variation in morbidity across the years included in our study [45]. It would obviously have been beneficial if effects of diet and life style could have been included in our analyses, but such data are not available in the Danish public health records.

Mostly of clinical relevance, antihypertensive treatment may itself negatively affect neurocognitive development of children, as suggested by recent studies that incriminated all three standard drugs in Denmark: methyldopa, labetalol and nifedip [48, 49], reviewed in Koren [50]. We did not have information on any antihypertensive treatments that may have been applied, so some of the observed effects may be due to a combination of pre-eclampsia and antihypertensive treatment. No study has yet disentangled the separate effects of condition and treatment on these comorbidities.

Finally, we would like to stress that the likelihood of our results having predictive value for other populations will depend on the neonatal and general obstetric support available at delivering units. We therefore expect that our results may be valid for other affluent societies, but that they are not directly transferable to developing countries.

## Supplementary Material

Supplementary Data

## References

[1] Steegers EA, von Dadelszen P, Duvekot JJ, Pijnenborg R Pre-eclampsia. Lancet 2010;376: 631–44. 20598363 10.1016/S0140-6736(10)60279-6

[2] Haddad B, Sibai BM. Expectant management in pregnancies with severe pre-eclampsia. Semin Perinatol 2009;33:143–51. 19464504 10.1053/j.semperi.2009.02.002

[3] Magee LA, von Dadelszen P. The management of severe hypertension. Semin Perinatol 2009;33:138–42. 19464503 10.1053/j.semperi.2009.02.001

[4] Koopmans CM, Bijlenga D, Groen H Induction of labour versus expectant monitoring for gestational hypertension or mild pre-eclampsia after 36 weeks' gestation (HYPITAT): a multicentre, open-label randomised controlled trial. Lancet 2009;374:979–88. 19656558 10.1016/S0140-6736(09)60736-4

[5] Langenveld J, Broekhuijsen K, van Baaren GJ Induction of labour versus expectant monitoring for gestational hypertension or mild pre-eclampsia between 34 and 37 weeks' gestation (HYPITAT-II): a multicentre, open-label randomised controlled trial. BMC Pregnancy Childbirth 2011;11:50. 21736705 10.1186/1471-2393-11-50 PMC3161905

[6] Moussa HN, Arian SE, Sibai BM. Management of hypertensive disorders in pregnancy. Women's Health 2014;10:385–404. 10.2217/whe.14.32 25259900

[7] Lykke JA, Paidas MJ, Langhoff-Roos J. Recurring complications in second pregnancy. Obstet Gynecol 2009;113:1217–24. 19461415 10.1097/AOG.0b013e3181a66f2d

[8] Lykke JA, Langhoff-Roos J, Sibai BM Hypertensive pregnancy disorders and subsequent cardiovascular morbidity and type 2 diabetes mellitus in the mother. Hypertension 2009;53:944–U102. 19433776 10.1161/HYPERTENSIONAHA.109.130765

[9] Roberts JM, Hubel CA. The two stage model of preeclampsia: variations on the theme. Placenta 2009;30:S32–7. 10.1016/j.placenta.2008.11.009 PMC2680383 19070896

[10] Clancy KBH. Inflammation, Reproduction, and the Goldilocks Principle In: Clancy KBH, Hinde K, Rutherford JN (eds). Building Babies: Primate Development in Proximate and Ultimate Perspective. New York, NY: Springer New York, 2013, 3–26.

[11] Robillard P-Y, Chaline J, Chaouat G, Hulsey TC Preeclampsia/eclampsia and the evolution of the human brain. Curr Anthropol 2003;44:130–5.

[12] Rosenberg KR, Trevathan WR. An anthropological perspective on the evolutionary context of preeclampsia in humans. J Reprod Immunol 2007;76:91–7. 17499857 10.1016/j.jri.2007.03.011

[13] Crosley EJ, Elliot MG, Christians JK, Crespi BJ Placental invasion, preeclampsia risk and adaptive molecular evolution at the origin of the great apes: evidence from genome-wide analyses. Placenta 2013;34:127–32. 23266291 10.1016/j.placenta.2012.12.001

[14] Pijnenborg R, Vercruysse L, Carter AM. Deep trophoblast invasion and spiral artery remodelling in the placental bed of the chimpanzee. Placenta 2011;32:400–8. 21459441 10.1016/j.placenta.2011.02.009

[15] Trivers RL. Parent-offspring conflict. Am Zool 1974;14:249–64.

[16] Haig D. Genetic conflicts in human-pregnancy. Q Rev Biol 1993;68:495–532. 8115596 10.1086/418300

[17] Haig D. Interbirth intervals: intrafamilial, intragenomic and intrasomatic conflict. Evol Med Public Health 2014;2014:12–7. 24480612 10.1093/emph/eou002 PMC3917425

[18] Moore T, Haig D. Genomic imprinting in mammalian development - a parental tug-of-war. Trends Genes 1991;7:45–9. 10.1016/0168-9525(91)90230-N 2035190

[19] Haig D. Genomic imprinting and the theory of parent-offspring conflict. Semin Dev Biol 1992;3:153–60.

[20] Queller DC. Theory of genomic imprinting conflict in social insects. BMC Evol Biol 2003;3:1. 12871603 10.1186/1471-2148-3-15 PMC194663

[21] Haig D, Úbeda F, Patten MM. Specialists and generalists: the sexual ecology of the genome. Cold Spring Harb Perspect Biol 2014;6:a017525. 25059710 10.1101/cshperspect.a017525 PMC4142971

[22] Kappil MA, Green BB, Armstrong DA Placental expression profile of imprinted genes impacts birth weight. Epigenetics 2015;10:842–9. 26186239 10.1080/15592294.2015.1073881 PMC4623427

[23] Moore GE, Ishida M, Demetriou C The role and interaction of imprinted genes in human fetal growth. Philos Trans R Soc B Biol Sci 2015;370:20140074. 10.1098/rstb.2014.0074 PMC4305174 25602077

[24] Hollegaard B, Byars SG, Lykke J, Boomsma JJ Parent-offspring conflict and the persistence of pregnancy-induced hypertension in modern humans. PLoS One 2013;8:e56821. 23451092 10.1371/journal.pone.0056821 PMC3581540

[25] von Dadelszen P, Magee LA, Taylor EL, Muir JC Maternal hypertension and neonatal outcome among small for gestational age infants. Obstet Gynecol 2005;106:335–9. 16055584 10.1097/01.AOG.0000171121.31564.14

[26] Chen XK, Wen SW, Smith G Pregnancy-induced hypertension and infant mortality: roles of birthweight centiles and gestational age. BJOG 2007;114:24–31. 17233856 10.1111/j.1471-0528.2006.01177.x

[27] Constância M, Hemberger M, Hughes J Placental-specific IGF-II is a major modulator of placental and fetal growth. Nature 2002;417:945–8. 12087403 10.1038/nature00819

[28] Byars SG, Stearns SC, Boomsma JJ. Opposite risk patterns for autism and schizophrenia are associated with normal variation in birth size: phenotypic support for hypothesized diametric gene-dosage effects. Proc R Soc B 2014;281:20140604. 10.1098/rspb.2014.0604 PMC4211440 25232142

[29] Staff AC, Dechend R, Redman CW. Review: preeclampsia, acute atherosis of the spiral arteries and future cardiovascular disease: two new hypotheses. Placenta 2013;34:S73–8. 23246096 10.1016/j.placenta.2012.11.022

[30] Maršál K, Persson PH, Larsen T Intrauterine growth curves based on ultrasonically estimated foetal weights. Acta Paediatr 1996;85:843–8. 8819552 10.1111/j.1651-2227.1996.tb14164.x

[31] Wu CS, Nohr EA, Bech BH, Vestergaard M Health of children born to mothers who had preeclampsia: a population-based cohort study. Am J Obstet Gynecol 2009;201:269.e1–269. 19733276 10.1016/j.ajog.2009.06.060

[32] Wilcox AJ, Skjaerven R. Birth weight and perinatal mortality: the effect of gestational age. Am J Public Health 1992;82:378–82. 1536353 10.2105/ajph.82.3.378 PMC1694365

[33] Mortensen LH, Helweg-Larsen K, Andersen AM. Socioeconomic differences in perinatal health and disease. Scand J Public Health 2011;39:110–4. 21775367 10.1177/1403494811405096

[34] Sutcliffe AG, Barnes J, Belsky J The health and development of children born to older mothers in the United Kingdom: observational study using longitudinal cohort data. BMJ 2012;345:e5116. 22915663 10.1136/bmj.e5116 PMC3424227

[35] Ødegård RA, Vatten LJ, Nilsen ST Preeclampsia and fetal growth. Obstet Gynecol 2000;96:950–5. 11084184

[36] Harcourt AH, Harvey PH, Larson SG Testis weight, body weight and breeding system in primates. Nature 1981;293:55–7. 7266658 10.1038/293055a0

[37] Samstein RM, Josefowicz SZ, Arvey A Extrathymic generation of regulatory T cells in placental mammals mitigates maternal-fetal conflict. Cell 2012;150:29–38. 22770213 10.1016/j.cell.2012.05.031 PMC3422629

[38] Mamun AA, Kinarivala MK, O'callaghan M Does hypertensive disorder of pregnancy predict offspring blood pressure at 21 years? Evidence from a birth cohort study. J Hum Hypertens 2012;26:288–94. 21509041 10.1038/jhh.2011.35

[39] McIntire DD, Bloom SL, Casey BM Birth weight in relation to morbidity and mortality among newborn infants. N Engl J Med 1999;340:1234–8. 10210706 10.1056/NEJM199904223401603

[40] Mathiasen R, Hansen BM, Andersen AMNN Gestational age and basic school achievements: a national follow-up study in Denmark. Pediatrics 2010;126:e1553–61. 21059721 10.1542/peds.2009-0829

[41] Morsing E, Marsal K. Pre-eclampsia- an additional risk factor for cognitive impairment at school age after intrauterine growth restriction and very preterm birth. Early Hum Dev 2014;90:99–101. 24388669 10.1016/j.earlhumdev.2013.12.002

[42] Bager P. Birth by caesarean section and wheezing, asthma, allergy, and intestinal disease. Clin Exp Allergy 2011;41:147–8. 21231975 10.1111/j.1365-2222.2010.03635.x

[43] Chang JJ, Muglia LJ, Macones GA. Association of early-onset pre-eclampsia in first pregnancy with normotensive second pregnancy outcomes: a population-based study. BJOG 2010;117:946–53. 20497414 10.1111/j.1471-0528.2010.02594.x PMC2884050

[44] Schmidt B, Davis PG, Asztalos EV Association between severe retinopathy of prematurity and nonvisual disabilities at age 5 years. JAMA 2014;311:523–5. 24496539 10.1001/jama.2013.282153

[45] Statistik D. *Danmark i tal, 1st edn (in Danish).* 2015, 40p. Statistics Denmark. ISBN-13: 9788750121671.

[46] Ovesen P, Rasmussen S, Kesmodel U. Effect of prepregnancy maternal overweight and obesity on pregnancy outcome. Obstet Gynecol 2011;118:305–12. 21775846 10.1097/AOG.0b013e3182245d49

[47] OECD. Divided We Stand: Why Inequality Keeps Rising. Paris: OECD Publishing, 2011.

[48] Pasker-de Jong PCM, Zielhuis GA, van Gelder MMHJ Antihypertensive treatment during pregnancy and functional development at primary school age in a historical cohort study. BJOG 2010;117:1080–6. 20477821 10.1111/j.1471-0528.2010.02568.x

[49] Magee LA, Abalos E, von Dadelszen P How to manage hypertension in pregnancy effectively. Br J Clin Pharmacol 2011;72:394–401. 21545480 10.1111/j.1365-2125.2011.04002.x PMC3175509

[50] Koren G. Systematic review of the effects of maternal hypertension in pregnancy and antihypertensive therapies on child neurocognitive development. Reprod Toxicol 2013;39:1–5 23542230 10.1016/j.reprotox.2013.03.006

