# Supplementary material for: Time from pre-eclampsia diagnosis to delivery affects future health prospects of children
Source: Evol Med Public Health. 2017 Feb 8;2017(1):53–66. doi: 10.1093/emph/eox004 (PMC5387983; doi:10.1093/emph/eox004)
Supplement: Supplementary Data [file eox004_supp.docx]

Figure S1. Average age of being diagnosed when exposed to either mild (no fill) or severe (sketched) preeclampsia, or being unexposed (grey). Upper and lower limits correspond to 1^st^ and 3^rd^ quartile (dashed lines) and black line is the mean.

Table S1. The ICD-8 and ICD-10 codes for the specific maternal preeclampsia diagnoses and the 14 major disease groups considered in this study.

| **Maternal preeclampsia and related diagnoses** | | | |
| --- | --- | --- | --- |
|  | **ICD-8** | **ICD-10^++^** | **Disease description** |
|  | 63703 | DO140 | Mild preeclampsia  (blood pressure ≥ 140/90 and 0.3 g/24 h protein in urine) |
|  | 63704 | DO141 | Severe preeclampsia (blood pressure ≥ 160/110 and/or 3 g/24 h protein in urine) |
|  | 63709 | DO149 | Unspecified preeclampsia |
|  | 63719 | DO15 | Eclampsia |
|  | 63799 | DO142 | HELLP Syndrome  (hemolysis elevated liver enzymes low platelets) |
| **The 14 disease categories of children’s diagnoses** | | | |
| **#** | **ICD-8** | **ICD-10^++^** | **Disease description** |
| 1 | 0-13999 | DA00-DB99 | Infection |
| 2 | 14000-23999 | DC00-DD48 | Neoplasm |
| 3 | 28000-28999 | DD50-DD89 | Blood |
| 4 | 24000-27999 | DE00-DE90 | Endocrine |
| 5 | 29000-31599 | DF00-DF99 | Behavior |
| 6 | 32000-35899 | DG00-DG99 | Nervous system |
| 7 | 36000-38999 | DH00-DH95 | Eye/ear |
| 8 | 39000-45899 | DI00-DI99 | Circulatory |
| 9 | 46000-51999 | DJ00-DJ99 | Respiratory |
| 10 | 52000-57799 | DK00-DK93 | Digestive system |
| 11 | 68000-70999 | DL00-DL99 | Skin |
| 12 | 71000-73799 | DM00-DM99 | Musculoskeletal |
| 13 | 58000-62999 | DN00-DN99 | Genitourinary |
| 14 | 73800-75999 | DQ00-DQ99 | Malformations |

**^++^**Classifications of disease groups can be found at [www.medinfo.dk/sks](http://www.medinfo.dk/sks)

Table S2. Hazard ratios (HRs) of the covariates used in the Cox regressions for the six delivery categories, of which we interpret the last five in detail in the manuscript. HRs above/below 1 indicate increased/decreased risks of being diagnosed with the given disease after experiencing either mild or severe preeclampsia relative to controls with normotensive pregnancies. 95% confidence intervals are in brackets. HRs in bold remained significant after FDR adjustment.

| **Extremely preterm (week 20-27)** | | | | | | | |
| --- | --- | --- | --- | --- | --- | --- | --- |
| **Disease groups** | **Birth year** | **Ponderal index** | **Sex** | **Parity** | **Father's age** | **Mother's age** | **Education** |
| infections | **0·80(0·70-0·93)** | 0·95(0·82-1·10) | 1·26(0·79-2·00) | 0·71(0·51-0·99) | 1·02(0·97-1·07) | 1·01(0·95-1·08) | 0·87(0·68-1·12) |
| neoplasm | 1·14(0·74-1·74) | 1·07(0·81-1·41) | 2·12(0·66-6·84) | 1·15(0·65-2·05) | 0·91(0·79-1·05) | **1·20(1·03-1·40)** | 0·78(0·44-1·39) |
| blood | **0·65(0·45-0·93)** | 1·01(0·71-1·44) | 0·46(0·12-1·76) | 1·20(0·62-2·30) | 0·93(0·80-1·08) | 1·06(0·90-1·24) | 1·07(0·59-1·94) |
| endocrine | **0·72(0·55-0·94)** | 1·00(0·78-1·28) | 0·87(0·39-1·95) | 0·82(0·45-1·50) | 1·03(0·93-1·13) | 0·96(0·85-1·08) | 1·45(0·95-2·20) |
| behavior | 1·26(0·95-1·67) | 1·05(0·89-1·24) | 1·06(0·53-2·10) | 0·90(0·55-1·47) | 1·04(0·97-1·12) | 0·95(0·86-1·05) | 1·00(0·68-1·46) |
| nervous system | 0·82(0·68-1·01) | 1·01(0·85-1·19) | 0·76(0·40-1·45) | 0·91(0·57-1·45) | 0·94(0·87-1·02) | 1·02(0·93-1·11) | 1·12(0·79-1·57) |
| eye/ear | 1·09(0·96-1·23) | **0·82(0·70-0·96)** | 0·72(0·50-1·03) | 1·03(0·81-1·30) | 1·00(0·96-1·04) | 0·99(0·94-1·05) | 0·95(0·78-1·16) |
| circulatory | 0·89(0·66-1·20) | 1·02(0·79-1·31) | 0·85(0·34-2·11) | 0·75(0·41-1·38) | 0·99(0·90-1·09) | 1·05(0·93-1·18) | 0·84(0·52-1·34) |
| respiratory | 0·96(0·86-1·07) | 0·94(0·85-1·05) | 0·81(0·58-1·14) | 1·11(0·90-1·38) | 0·98(0·95-1·02) | 0·98(0·93-1·03) | 0·97(0·81-1·16) |
| digestive system | 1·07(0·93-1·23) | 0·92(0·78-1·07) | **0·46(0·30-0·73)** | 1·12(0·85-1·46) | 0·98(0·93-1·02) | 1·02(0·96-1·08) | 1·12(0·90-1·40) |
| skin | 1·13(0·80-1·62) | **1·27(1·07-1·50)** | **0·20(0·05-0·71)** | 1·22(0·66-2·24) | 0·97(0·87-1·08) | 1·01(0·88-1·16) | 0·99(0·60-1·63) |
| musculoskeletal | 1·38(0·98-1·92) | 0·94(0·75-1·18) | **2·39(1·14-5·00)** | 1·09(0·72-1·65) | 0·98(0·90-1·07) | 1·01(0·91-1·12) | 0·89(0·60-1·32) |
| genitourinary | 1·14(0·86-1·50) | 0·95(0·75-1·21) | 0·62(0·28-1·38) | 0·91(0·53-1·56) | 0·93(0·85-1·03) | 1·01(0·91-1·12) | 0·73(0·48-1·11) |
| malformations | 0·98(0·88-1·10) | **0·78(0·66-0·93)** | 1·15(0·81-1·63) | 0·89(0·70-1·12) | 0·99(0·95-1·03) | 1·03(0·98-1·09) | 0·94(0·77-1·14) |
| **Very preterm (week 28-33)** | | | | | | | |
| infections | **0·91(0·88-0·95)** | **0·92(0·89-0·96)** | 0·89(0·79-1·01) | 1·08(1·00-1·18) | 1·01(0·99-1·02) | **0·98(0·96-1·00)** | **0·88(0·82-0·94)** |
| neoplasm | **1·25(1·10-1·42)** | 0·98(0·90-1·07) | 1·30(0·96-1·76) | 0·97(0·78-1·20) | 1·03(0·99-1·06) | 0·98(0·94-1·02) | 1·00(0·85-1·17) |
| blood | **0·82(0·73-0·92)** | 0·90(0·80-1·01) | 0·71(0·50-1·02) | 1·15(0·94-1·41) | 1·03(0·99-1·07) | 1·00(0·95-1·05) | **0·79(0·66-0·94)** |
| endocrine | **0·85(0·80-0·91)** | **0·88(0·82-0·94)** | 1·11(0·92-1·33) | 1·01(0·89-1·15) | 1·01(0·99-1·03) | 0·99(0·96-1·01) | **0·87(0·79-0·97)** |
| behavior | 1·10(1·00-1·21) | 0·95(0·89-1·02) | **0·71(0·56-0·90)** | 0·93(0·78-1·11) | 1·00(0·97-1·03) | 0·98(0·95-1·02) | 0·90(0·80-1·02) |
| nervous system | 1·01(0·95-1·09) | **0·89(0·83-0·95)** | **0·79(0·64-0·97)** | 1·13(0·99-1·29) | 0·99(0·97-1·02) | 0·99(0·96-1·01) | 0·94(0·84-1·04) |
| eye/ear | **1·09(1·04-1·14)** | **0·92(0·89-0·96)** | 0·88(0·77-0·99) | 1·02(0·94-1·12) | 1·00(0·98-1·01) | 1·00(0·98-1·02) | 0·94(0·88-1·01) |
| circulatory | 0·96(0·84-1·09) | **0·87(0·77-0·98)** | 1·22(0·87-1·73) | 1·21(0·97-1·52) | 0·99(0·95-1·03) | 0·97(0·93-1·02) | 0·92(0·77-1·11) |
| respiratory | **1·07(1·04-1·10)** | **0·94(0·91-0·96)** | **0·76(0·69-0·84)** | **1·12(1·05-1·20)** | 0·99(0·98-1·00) | 0·99(0·98-1·01) | 0·97(0·92-1·02) |
| digestive system | 1·01(0·96-1·06) | **0·92(0·88-0·96)** | **0·54(0·47-0·62)** | 0·96(0·88-1·06) | 1·00(0·98-1·01) | 1·01(0·99-1·03) | **0·92(0·85-0·98)** |
| skin | **1·16(1·07-1·26)** | 0·97(0·92-1·03) | 0·96(0·78-1·18) | 1·07(0·93-1·23) | 1·03(1·01-1·06) | 0·98(0·95-1·01) | 1·00(0·89-1·12) |
| musculoskeletal | **1·34(1·24-1·45)** | 1·02(0·97-1·06) | 1·07(0·91-1·27) | 1·01(0·90-1·14) | 1·00(0·98-1·02) | 0·99(0·97-1·01) | 1·00(0·92-1·10) |
| genitourinary | 0·98(0·91-1·06) | 0·96(0·91-1·01) | **0·78(0·65-0·95)** | 1·05(0·92-1·20) | 0·99(0·96-1·01) | 1·00(0·98-1·03) | 1·05(0·95-1·16) |
| malformations | **1·07(1·03-1·12)** | **0·90(0·87-0·94)** | **0·77(0·67-0·88)** | 0·98(0·89-1·07) | 1·00(0·99-1·02) | 0·99(0·97-1·01) | 0·95(0·89-1·02) |
| **Preterm (week 34-36)** | | | | | | | |
| infections | **1·05(1·02-1·08)** | **0·97(0·95-1·00)** | **0·88(0·81-0·94)** | 1·01(0·96-1·07) | 1·01(1·00-1·01) | **0·98(0·97-0·99)** | 0·96(0·93-1·00) |
| neoplasm | **1·15(1·07-1·24)** | 1·01(0·96-1·07) | **1·52(1·28-1·80)** | 0·90(0·79-1·03) | 0·98(0·96-1·00) | 1·02(0·99-1·04) | 1·09(1·00-1·20) |
| blood | **0·89(0·83-0·96)** | 0·93(0·88-0·99) | 0·93(0·76-1·15) | 0·98(0·84-1·14) | 1·00(0·98-1·03) | 0·98(0·95-1·01) | 0·94(0·84-1·05) |
| endocrine | 0·96(0·92-1·00) | **0·89(0·86-0·92)** | **1·24(1·11-1·39)** | 1·00(0·92-1·08) | 1·01(0·99-1·02) | 0·99(0·98-1·01) | **0·91(0·86-0·97)** |
| behavior | **1·18(1·11-1·25)** | **0·95(0·91-0·99)** | 0·97(0·85-1·10) | 0·97(0·89-1·06) | **1·02(1·01-1·04)** | **0·97(0·95-0·99)** | **0·88(0·82-0·94)** |
| nervous system | 1·04(0·99-1·09) | 0·98(0·94-1·01) | 1·04(0·91-1·18) | 1·02(0·94-1·12) | 1·01(1·00-1·03) | **0·98(0·96-1·00)** | 1·01(0·94-1·08) |
| eye/ear | **1·07(1·04-1·10)** | **0·95(0·93-0·97)** | **0·74(0·69-0·80)** | 1·01(0·96-1·07) | 1·00(0·99-1·01) | 0·99(0·98-1·00) | 0·97(0·93-1·00) |
| circulatory | **1·11(1·02-1·21)** | 0·95(0·89-1·00) | 1·06(0·87-1·29) | 1·00(0·86-1·15) | 1·01(0·99-1·03) | 0·99(0·96-1·02) | 1·03(0·93-1·14) |
| respiratory | **1·04(1·02-1·06)** | **0·98(0·97-1·00)** | **0·76(0·72-0·80)** | **1·09(1·05-1·13)** | 1·00(0·99-1·00) | **0·98(0·98-0·99)** | 0·99(0·96-1·02) |
| digestive system | **1·04(1·01-1·07)** | **0·93(0·91-0·96)** | **0·68(0·63-0·73)** | 1·02(0·97-1·07) | 1·00(0·99-1·01) | 0·99(0·98-1·00) | 0·96(0·93-1·00) |
| skin | **1·11(1·06-1·16)** | 1·02(0·99-1·05) | 0·92(0·83-1·03) | 1·04(0·96-1·12) | 1·00(0·99-1·02) | **0·98(0·96-0·99)** | 1·02(0·96-1·08) |
| musculoskeletal | **1·36(1·31-1·42)** | 1·00(0·97-1·02) | 1·07(0·99-1·16) | 1·00(0·95-1·06) | 1·00(0·99-1·01) | 0·99(0·98-1·00) | 1·01(0·97-1·06) |
| genitourinary | **1·04(1·00-1·08)** | 0·98(0·95-1·01) | **0·83(0·76-0·91)** | 0·93(0·87-1·00) | 1·00(0·99-1·01) | **0·98(0·97-0·99)** | 0·99(0·94-1·04) |
| malformations | **1·07(1·04-1·10)** | **0·91(0·89-0·93)** | **0·75(0·69-0·81)** | 1·01(0·96-1·07) | 1·00(0·99-1·01) | 1·00(0·99-1·02) | 1·01(0·97-1·06) |
| **Early term (week 37-38)** | | | | | | | |
| infections | **1·14(1·13-1·16)** | **0·97(0·96-0·98)** | **0·89(0·86-0·92)** | **1·04(1·02-1·07)** | 1·00(0·99-1·00) | **0·99(0·98-0·99)** | **0·96(0·94-0·97)** |
| neoplasm | **1·14(1·10-1·18)** | 1·01(0·98-1·03) | **1·49(1·38-1·61)** | 1·00(0·95-1·06) | 1·00(0·99-1·00) | 1·00(0·99-1·01) | 1·04(1·00-1·08) |
| blood | **0·96(0·92-0·99)** | **0·94(0·91-0·96)** | 0·99(0·89-1·10) | **1·12(1·04-1·20)** | 1·01(1·00-1·02) | 0·98(0·97-1·00) | 0·95(0·90-1·00) |
| endocrine | **1·17(1·14-1·20)** | **0·92(0·91-0·94)** | **1·36(1·28-1·44)** | 1·04(1·00-1·09) | 1·00(0·99-1·01) | **0·99(0·98-1·00)** | **0·92(0·89-0·94)** |
| behavior | **1·23(1·20-1·27)** | **0·94(0·93-0·96)** | **0·87(0·82-0·92)** | 1·04(1·00-1·09) | 1·01(1·00-1·01) | **0·97(0·96-0·98)** | **0·90(0·87-0·93)** |
| nervous system | **1·10(1·07-1·12)** | **0·96(0·95-0·98)** | 0·98(0·92-1·05) | 1·03(0·99-1·08) | 1·01(1·00-1·01) | **0·98(0·97-0·99)** | **0·94(0·91-0·98)** |
| eye/ear | **1·07(1·06-1·09)** | **0·97(0·96-0·98)** | **0·76(0·73-0·79)** | 1·01(0·99-1·04) | 1·00(0·99-1·00) | **0·99(0·98-0·99)** | **0·98(0·96-0·99)** |
| circulatory | **1·16(1·11-1·22)** | 1·00(0·97-1·03) | 0·95(0·86-1·05) | 1·04(0·97-1·11) | 0·99(0·98-1·00) | 1·00(0·99-1·02) | 0·98(0·93-1·03) |
| respiratory | **1·07(1·06-1·08)** | **0·99(0·98-1·00)** | **0·74(0·72-0·76)** | **1·09(1·07-1·11)** | **0·99(0·99-1·00)** | **0·98(0·98-0·99)** | **0·96(0·95-0·98)** |
| digestive system | **1·05(1·04-1·07)** | **0·96(0·95-0·97)** | **0·76(0·73-0·78)** | 1·01(0·99-1·04) | 1·00(0·99-1·00) | **0·99(0·99-1·00)** | **0·95(0·93-0·97)** |
| skin | **1·18(1·16-1·21)** | 1·00(0·98-1·01) | **0·93(0·89-0·98)** | 1·01(0·98-1·05) | 1·00(0·99-1·00) | 0·99(0·98-1·00) | 0·99(0·96-1·01) |
| musculoskeletal | **1·33(1·30-1·35)** | 1·00(0·99-1·01) | 1·02(0·99-1·06) | 0·98(0·95-1·00) | **0·99(0·99-1·00)** | 1·00(0·99-1·00) | 1·00(0·98-1·02) |
| genitourinary | **1·08(1·06-1·10)** | 0·99(0·98-1·00) | **0·88(0·84-0·92)** | 0·97(0·94-1·00) | 0·99(0·99-1·00) | 0·99(0·99-1·00) | **0·97(0·94-0·99)** |
| malformations | **1·10(1·08-1·11)** | **0·96(0·95-0·97)** | **0·71(0·68-0·74)** | **0·94(0·92-0·97)** | 1·00(0·99-1·00) | 1·01(1·00-1·01) | **0·97(0·95-0·99)** |

Table S2 continued.

| **Full term (week 39-40)** | | | | | | | |
| --- | --- | --- | --- | --- | --- | --- | --- |
| **Disease groups** | **Birth year** | **Ponderal index** | **Sex** | **Parity** | **Father's age** | **Mother's age** | **Education** |
| infections | **1·15(1·14-1·15)** | **0·97(0·97-0·98)** | **0·92(0·91-0·94)** | 0·99(0·98-1·01) | 1·00(1·00-1·00) | **0·99(0·98-0·99)** | **0·96(0·95-0·97)** |
| neoplasm | **1·14(1·12-1·16)** | **1·02(1·00-1·03)** | **1·45(1·39-1·50)** | 0·99(0·96-1·02) | 1·00(0·99-1·00) | 1·00(0·99-1·00) | **1·02(1·00-1·04)** |
| blood | 1·00(0·98-1·02) | **0·95(0·93-0·96)** | 0·95(0·90-1·01) | **1·06(1·02-1·10)** | 1·00(1·00-1·01) | **0·98(0·97-0·99)** | 0·97(0·95-1·00) |
| endocrine | **1·26(1·24-1·27)** | **0·95(0·94-0·96)** | **1·65(1·59-1·70)** | 1·02(0·99-1·04) | 1·00(0·99-1·00) | **0·98(0·98-0·99)** | **0·90(0·89-0·92)** |
| behavior | **1·29(1·27-1·31)** | **0·96(0·95-0·97)** | **0·86(0·83-0·89)** | **1·05(1·03-1·08)** | 1·00(1·00-1·00) | **0·97(0·97-0·98)** | **0·91(0·89-0·92)** |
| nervous system | **1·12(1·11-1·14)** | **0·97(0·96-0·98)** | 0·99(0·95-1·02) | **1·05(1·02-1·07)** | 1·00(1·00-1·00) | **0·98(0·98-0·99)** | **0·95(0·93-0·97)** |
| eye/ear | **1·09(1·09-1·10)** | **0·97(0·97-0·98)** | **0·79(0·77-0·80)** | **0·98(0·96-0·99)** | 1·00(1·00-1·00) | **0·99(0·99-0·99)** | **0·97(0·96-0·98)** |
| circulatory | **1·21(1·18-1·24)** | 0·99(0·97-1·00) | 1·00(0·95-1·04) | 1·01(0·98-1·05) | 1·00(0·99-1·00) | 0·99(0·98-1·00) | 0·98(0·96-1·00) |
| respiratory | **1·08(1·07-1·08)** | **0·98(0·98-0·99)** | **0·76(0·75-0·77)** | **1·04(1·03-1·05)** | 1·00(1·00-1·00) | **0·98(0·98-0·98)** | **0·96(0·96-0·97)** |
| digestive system | **1·06(1·05-1·07)** | **0·98(0·97-0·98)** | **0·81(0·80-0·82)** | **1·03(1·02-1·04)** | 1·00(1·00-1·00) | **0·99(0·98-0·99)** | **0·95(0·94-0·96)** |
| skin | **1·19(1·18-1·20)** | 1·00(1·00-1·01) | **0·89(0·87-0·91)** | 1·00(0·98-1·02) | 1·00(1·00-1·00) | **0·99(0·98-0·99)** | **0·96(0·95-0·98)** |
| musculoskeletal | **1·33(1·32-1·34)** | 1·00(1·00-1·01) | **1·10(1·08-1·12)** | 0·99(0·98-1·00) | 1·00(1·00-1·00) | **0·99(0·99-0·99)** | **0·99(0·98-1·00)** |
| genitourinary | **1·10(1·09-1·11)** | **0·99(0·98-1·00)** | **0·98(0·95-1·00)** | **0·96(0·95-0·98)** | 1·00(0·99-1·00) | **0·99(0·99-0·99)** | **0·98(0·96-0·99)** |
| malformations | **1·10(1·09-1·11)** | **0·97(0·97-0·98)** | **0·71(0·70-0·73)** | **0·91(0·90-0·92)** | 1·00(1·00-1·00) | 1·00(1·00-1·01) | **0·98(0·97-0·99)** |
| **Post term (week 41-44)** | | | | | | | |
| infections | **1·13(1·12-1·14)** | **0·98(0·97-0·99)** | **0·91(0·89-0·93)** | 0·98(0·96-1·00) | 1·00(1·00-1·00) | **0·98(0·98-0·99)** | **0·97(0·95-0·98)** |
| neoplasm | **1·14(1·11-1·16)** | 1·01(1·00-1·03) | **1·50(1·42-1·58)** | 1·00(0·96-1·04) | 1·00(0·99-1·00) | 1·00(0·99-1·01) | 1·03(1·00-1·06) |
| blood | 1·00(0·97-1·03) | 0·99(0·96-1·01) | 0·94(0·87-1·02) | 1·04(0·98-1·10) | 1·00(0·99-1·01) | **0·98(0·97-1·00)** | 0·99(0·95-1·04) |
| endocrine | **1·26(1·24-1·28)** | **0·95(0·94-0·97)** | **1·63(1·56-1·70)** | 1·00(0·97-1·03) | 1·00(0·99-1·00) | **0·98(0·98-0·99)** | **0·89(0·87-0·91)** |
| behavior | **1·30(1·27-1·33)** | **0·96(0·95-0·97)** | **0·85(0·81-0·88)** | **1·05(1·02-1·08)** | **1·01(1·00-1·01)** | **0·97(0·96-0·98)** | **0·90(0·88-0·92)** |
| nervous system | **1·11(1·09-1·13)** | **0·97(0·96-0·98)** | 0·97(0·92-1·01) | 0·98(0·95-1·02) | 1·00(1·00-1·01) | **0·99(0·98-1·00)** | **0·94(0·91-0·96)** |
| eye & ear | **1·10(1·09-1·11)** | **0·97(0·96-0·98)** | **0·79(0·77-0·81)** | **0·96(0·94-0·98)** | 1·00(1·00-1·00) | **0·99(0·98-0·99)** | **0·97(0·95-0·98)** |
| circulatory | **1·21(1·17-1·25)** | 1·00(0·98-1·01) | **0·90(0·84-0·96)** | 1·03(0·98-1·08) | 1·00(0·99-1·00) | 0·99(0·98-1·00) | 0·98(0·94-1·01) |
| respiratory | **1·08(1·07-1·09)** | **0·98(0·98-0·99)** | **0·76(0·75-0·78)** | **1·02(1·01-1·04)** | 1·00(1·00-1·00) | **0·98(0·98-0·98)** | **0·96(0·95-0·97)** |
| digestive system | **1·07(1·06-1·08)** | **0·98(0·97-0·99)** | **0·81(0·79-0·83)** | 1·02(1·00-1·04) | 1·00(1·00-1·00) | **0·99(0·98-0·99)** | **0·96(0·95-0·97)** |
| skin | **1·20(1·18-1·22)** | 1·00(0·99-1·01) | **0·88(0·85-0·91)** | 1·00(0·98-1·03) | 1·00(1·00-1·01) | **0·99(0·98-0·99)** | 0·99(0·97-1·00) |
| musculoskeletal | **1·33(1·32-1·35)** | 1·00(0·99-1·00) | **1·10(1·07-1·12)** | 1·00(0·98-1·02) | 1·00(0·99-1·00) | **0·99(0·99-1·00)** | **0·98(0·97-1·00)** |
| genitourinary | **1·12(1·11-1·14)** | 0·99(0·99-1·00) | **0·95(0·92-0·98)** | **0·97(0·95-0·99)** | 1·00(1·00-1·00) | **0·98(0·98-0·99)** | 0·99(0·98-1·01) |
| malformations | **1·10(1·08-1·11)** | **0·98(0·97-0·99)** | **0·71(0·69-0·73)** | **0·90(0·88-0·92)** | 1·00(1·00-1·01) | 1·00(1·00-1·01) | **0·95(0·94-0·97)** |

Table S3. Hazard ratios of disease diagnoses (from birth up to 30 years of age) within the 14 disease group depending on whether offspring were born to mothers with mild or severe preeclampsia, categorized by gestation category. Values are hazard ratios relative to offspring of mothers with normotensive pregnancies (HR < 1 when risk is reduced and HR > 1 when risk is increased) based on Cox regressions (95% confidence intervals in brackets). HRs in bold remained significant after false discovery rate adjustment.

| **Diseases** | **Week 20 - 27** | | **Week 28 - 33** | | **Week 34 - 36** | | **Week 37 - 38** | | **Week 39 - 40** | | **Week 41 - 44** | |
| --- | --- | --- | --- | --- | --- | --- | --- | --- | --- | --- | --- | --- |
|  | **Preeclampsia** | | **Preeclampsia** | | **Preeclampsia** | | **Preeclampsia** | | **Preeclampsia** | | **Preeclampsia** | |
|  | **Mild** | **Severe** | **Mild** | **Severe** | **Mild** | **Severe** | **Mild** | **Severe** | **Mild** | **Severe** | **Mild** | **Severe** |
| **infections** | NA | 0·44  (0·17-1·15) | 1·16  (0·85-1·57) | 0·94  (0·77-1·14) | 1·15  (0·97-1·35) | 0·97  (0·81-1·16) | **1·26 (1·16-1·36)** | **1·16 (1·00-1·35)** | **1·22 (1·16-1·29)** | 1·06 (0·90-1·23) | **1·21 (1·12-1·31)** | **1·36 (1·05-1·77)** |
| **neoplasm** | NA | 1·12  (0·18-7·07) | 2·22  (1·17-4·19) | 1·58  (1·00-2·51) | 0·75  (0·49-1·16) | 1·09  (0·75-1·60) | 1·04 (0·86-1·26) | 1·18 (0·85-1·64) | 1·05 (0·94-1·18) | 1·03 (0·75-1·42) | 0·95 (0·81-1·12) | 0·92 (0·51-1·68) |
| **blood** | NA | NA | 1·60  (0·74-3·44) | 1·35  (0·80-2·28) | 1·14  (0·73-1·77) | 0·99  (0·62-1·59) | 0·98  (0·75-1·30) | 1·13 (0·71-1·82) | 1·16 (0·99-1·36) | 1·04 (0·65-1·64) | 1·21 (0·96-1·52) | 1·44 (0·68-3·05) |
| **endocrine** | NA | 1·82  (0·56-5·84) | 1·49  (0·95-2·33) | 1·46  (1·09-1·96) | **1·48  (1·17-1·87)** | **1·78 (1·43-2·23)** | **1·51 (1·34-1·71)** | **1·51 (1·21-1·88)** | **1·55 (1·44-1·68)** | **1·37 (1·09-1·72)** | **1·65 (1·45-1·88)** | 1·40 (0·89-2·21) |
| **mental** | NA | 0·95  (0·27-3·29) | 1·47  (0·84-2·57) | 1·08  (0·73-1·59) | **1·47 (1·14-1·90)** | 1·03  (0·76-1·39) | **1·19 (1·04-1·37)** | 0·91 (0·69-1·19) | **1·30 (1·20-1·42)** | **1·46 (1·18-1·81)** | 1·15 (1·02-1·30) | 1·01 (0·64-1·57) |
| **nervous system** | NA | 0·82  0·27-2·51) | 0·73  (0·43-1·26) | 0·73  (0·53-1·01) | 1·23 (0·93-1·63) | 0·96  (0·71-1·32) | 1·18 (1·02-1·36) | 1·20 (0·92-1·56) | **1·34 (1·22-1·46)** | **1·38 (1·08-1·75)** | **1·27 (1·11-1·45)** | **1·74 (1·17-2·59)** |
| **eye /ear** | NA | 0·60  (0·30-1·23) | 1·11  (0·81-1·52) | 0·90  (0·74-1·11) | 1·15 (0·97-1·36) | 0·96  (0·80-1·15) | **1·20  (1·10-1·30)** | **1·20 (1·03-1·40)** | **1·19 (1·13-1·26)** | 1·15 (0·99-1·34) | **1·14 (1·05-1·24)** | 1·21 (0·92-1·59) |
| **circulatory** | NA | 0·71  (0·13-4·03) | 1·38  (0·64-2·95) | 1·04  (0·61-1·78) | 1·57  (1·04-2·38) | 1·25  (0·78-1·99) | 1·13 (0·91-1·41) | 1·09 (0·73-1·63) | **1·37 (1·20-1·56)** | **1·11 (0·75-1·63)** | **1·29 (1·07-1·55)** | 1·45 (0·79-2·65) |
| **respiratory** | NA | 0·56  (0·30-1·05) | 1·14  (0·90-1·45) | 0·99  (0·85-1·15) | **1·19  (1·06-1·35)** | 1·04  (0·92-1·18) | **1·24 (1·17-1·32)** | **1·19 (1·07-1·33)** | **1·25 (1·21-1·30)** | 1·12 (1·00-1·25) | **1·20 (1·14-1·27)** | **1·31 (1·08-1·59)** |
| **digestive** | NA | 0·82  (0·42-1·62) | 1·43  (1·03-1·98) | 1·16  (0·94-1·44) | 1·10 (0·93-1·30) | 1·12  (0·95-1·33) | **1·19 (1·10-1·29)** | **1·20 (1·04-1·40)** | **1·20 (1·14-1·26)** | **1·23 (1·07-1·41)** | **1·25 (1·16-1·35)** | 1·16 (0·89-1·52) |
| **skin** | NA | 1·75  (0·41-7·43) | 0·61  (0·31-1·21) | 0·89  (0·62-1·27) | 1·20 (0·94-1·53) | 1·13  (0·87-1·46) | 1·13 (1·01-1·27) | 1·07 (0·86-1·33) | **1·22 (1·14-1·31)** | 1·20 (0·99-1·46) | 1·07 (0·97-1·19) | 1·26 (0·89-1·77) |
| **musculoskeletal** | NA | 0·89  (0·20-4·01) | 1·58  (1·09-2·29) | 1·07  (0·82-1·40) | 1·05 (0·88-1·27) | 0·87  (0·71-1·07) | 1·05 (0·96-1·14) | 1·06 (0·91-1·24) | **1·14 (1·09-1·20)** | 1·06 (0·92-1·23) | **1·15 (1·07-1·24)** | 1·12 (0·87-1·44) |
| **genitourinary** | NA | 0·69  (0·17-2·88) | 1·18  (0·75-1·87) | 1·00  (0·73-1·35) | 1·15 (0·93-1·42) | 1·07  (0·85-1·34) | 1·11 (1·00-1·23) | 1·16 (0·96-1·39) | **1·12 (1·06-1·20)** | 0·96 (0·80-1·16) | **1·15 (1·04-1·26)** | 1·20 (0·88-1·64) |
| **malformations** | 0·91  (0·12-6·84) | 0·75  (0·41-1·37) | 1·05  (0·76-1·45) | 0·99  (0·81-1·21) | **1·27 (1·07-1·51)** | 0·95  (0·79-1·15) | **1·16 (1·06-1·27)** | 0·95 (0·80-1·14) | **1·12 (1·05-1·19)** | 1·16 (0·99-1·37) | **1·11 (1·02-1·21)** | 1·25 (0·94-1·67) |

Table S4. Absolute risks (%) of being diagnosed when exposed to mild or severe preeclampsia compared to the unexposed group (controls). Red arrows (🡹) indicate increased risk and green arrows (🡻) indicate decreased risk, all compared to unexposed. Risks are ranked in descending order and per gestation category.

| **Very preterm (week 28-33)** | | | | |
| --- | --- | --- | --- | --- |
|  | **Preeclampsia** | | | |
|  | Mild | | Severe | |
| neoplasm | 104,03 | 🡹 | 44,18 | 🡹 |
| blood | 79,79 | 🡹 | 42,94 | 🡹 |
| endocrine | 45,54 | 🡹 | 42,53 | 🡹 |
| circulatory | 66,40 | 🡹 | 26,78 | 🡹 |
| musculoskeletal | 65,37 | 🡹 | 13,43 | 🡹 |
| malformations | 16,98 | 🡹 | 12,19 | 🡹 |
| infections | 33,07 | 🡹 | 10,11 | 🡹 |
| respiratory | 16,18 | 🡹 | 7,76 | 🡹 |
| digestive system | 22,74 | 🡹 | 4,39 | 🡹 |
| eye & ear | 21,19 | 🡹 | 4,20 | 🡹 |
| genitourinary system | 28,40 | 🡹 | 3,63 | 🡹 |
| behavior | 34,54 | 🡹 | 2,20 | 🡹 |
| nervous system | -2,24 | 🡻 | 0,18 | 🡹 |
| skin | -36,47 | 🡻 | -10,21 | 🡻 |
| *U* = 52, Z-score = 2.09, p = 0.04 | | | | |
| **Preterm (week 34-36)** | | | | |
|  | **Preeclampsia** | | | |
|  | Mild | | Severe | |
| endocrine | 76,17 | 🡹 | 110,63 | 🡹 |
| neoplasm | 2,94 | 🡹 | 46,38 | 🡹 |
| circulatory | 69,69 | 🡹 | 31,48 | 🡹 |
| blood | 47,44 | 🡹 | 30,32 | 🡹 |
| digestive system | 23,50 | 🡹 | 25,52 | 🡹 |
| behavior | 78,65 | 🡹 | 24,83 | 🡹 |
| skin | 34,82 | 🡹 | 20,05 | 🡹 |
| genitourinary system | 32,04 | 🡹 | 15,43 | 🡹 |
| nervous system | 43,80 | 🡹 | 11,84 | 🡹 |
| respiratory | 21,71 | 🡹 | 8,11 | 🡹 |
| malformations | 33,11 | 🡹 | 5,55 | 🡹 |
| eye & ear | 23,82 | 🡹 | 5,35 | 🡹 |
| infections | 22,78 | 🡹 | 5,01 | 🡹 |
| musculoskeletal | 24,22 | 🡹 | 2,53 | 🡹 |
| *U* = 55.5, Z-score = 1.93, p = 0.054 | | | | |

| **Early term (week 37-38)** | | | | |
| --- | --- | --- | --- | --- |
|  | **Preeclampsia** | | | |
|  | Mild | | Severe | |
| endocrine | 76,47 | 🡹 | 79,10 | 🡹 |
| neoplasm | 34,57 | 🡹 | 47,68 | 🡹 |
| nervous system | 46,67 | 🡹 | 47,59 | 🡹 |
| circulatory | 55,72 | 🡹 | 43,14 | 🡹 |
| genitourinary system | 41,67 | 🡹 | 41,96 | 🡹 |
| digestive system | 40,30 | 🡹 | 38,06 | 🡹 |
| musculoskeletal | 34,63 | 🡹 | 33,60 | 🡹 |
| eye & ear | 31,25 | 🡹 | 31,21 | 🡹 |
| behavior | 65,09 | 🡹 | 27,28 | 🡹 |
| infections | 33,01 | 🡹 | 24,58 | 🡹 |
| blood | 9,61 | 🡹 | 23,31 | 🡹 |
| respiratory | 28,85 | 🡹 | 23,19 | 🡹 |
| skin | 31,27 | 🡹 | 20,99 | 🡹 |
| malformations | 25,38 | 🡹 | 7,07 | 🡹 |
| *U* = 80, Z-score = 0.80, p = 0.42 | | | | |
| **Full term (week 39-40)** | | | | |
|  | **Preeclampsia** | | | |
|  | Mild | | Severe | |
| behavior | 75,30 | 🡹 | 87,24 | 🡹 |
| endocrine | 91,24 | 🡹 | 63,79 | 🡹 |
| nervous system | 63,37 | 🡹 | 61,55 | 🡹 |
| digestive system | 49,38 | 🡹 | 44,21 | 🡹 |
| skin | 46,52 | 🡹 | 37,68 | 🡹 |
| circulatory | 84,15 | 🡹 | 33,41 | 🡹 |
| musculoskeletal | 48,13 | 🡹 | 30,90 | 🡹 |
| eye & ear | 34,40 | 🡹 | 30,26 | 🡹 |
| malformations | 20,82 | 🡹 | 28,95 | 🡹 |
| neoplasm | 39,90 | 🡹 | 25,84 | 🡹 |
| blood | 43,42 | 🡹 | 24,29 | 🡹 |
| respiratory | 34,00 | 🡹 | 21,85 | 🡹 |
| genitourinary system | 50,94 | 🡹 | 20,68 | 🡹 |
| infections | 31,49 | 🡹 | 14,85 | 🡹 |
| *U* = 53, Z-score = 2.04, p = 0.04 | | | | |
| **Post term (week 41-44)** | | | | |
|  | **Preeclampsia** | | | |
|  | Mild | | Severe | |
| nervous system | 61,82 | 🡹 | 109,65 | 🡹 |
| circulatory | 91,31 | 🡹 | 95,42 | 🡹 |
| blood | 53,89 | 🡹 | 76,06 | 🡹 |
| skin | 36,83 | 🡹 | 52,98 | 🡹 |
| endocrine | 92,40 | 🡹 | 50,82 | 🡹 |
| infections | 34,86 | 🡹 | 50,58 | 🡹 |
| genitourinary system | 54,13 | 🡹 | 48,61 | 🡹 |
| behavior | 71,09 | 🡹 | 44,51 | 🡹 |
| malformations | 23,83 | 🡹 | 44,34 | 🡹 |
| respiratory | 31,91 | 🡹 | 43,37 | 🡹 |
| digestive system | 58,90 | 🡹 | 42,86 | 🡹 |
| musculoskeletal | 55,17 | 🡹 | 42,42 | 🡹 |
| eye & ear | 29,76 | 🡹 | 38,71 | 🡹 |
| neoplasm | 37,83 | 🡹 | 22,67 | 🡹 |
| *U* = 96, Z-score = -0.07, p = 0.94 | | | | |

Table S5. Changes in Hazard ratios (HR) per day of exposure to mild or severe preeclampsia as estimated in two separate multiple regression models after adjustment for the same categorical effects and potentially confounding predictor variables (table 1) across the five windows of gestational length. See legend of Figure 3 for further overall rationale, which is supplemented here by a complete list of sample sizes, hazard ratios (with 95% CI in brackets) and p-values with those in red being significant before FDR adjustment.
